# Unravelling the Allergy Label: A Case of Successful Multi-drug Allergy De-labelling in a Patient

**DOI:** 10.7759/cureus.110983

**Published:** 2026-06-16

**Authors:** Angeliki Vassila, Ying Teo, Michael A Jones

**Affiliations:** 1 Department of Dermatology, University Hospital Southampton NHS Foundation Trust, Southampton, GBR; 2 Department of Cutaneous Allergy, St John's Institute of Dermatology, Guy's Hospital, London, GBR; 3 Department of Clinical Experimental Sciences, Faculty of Medicine, University of Southampton, Southampton, GBR

**Keywords:** corneal transplants, drug allergy, mucositis, mycoplasma pneumonia, rash, steven-johnson syndrome

## Abstract

We report the case of a 23-year-old woman with a pre-existing penicillin allergy label from childhood who presented with severe bilateral conjunctivitis and oral mucositis following a prodromal respiratory illness. Her symptoms worsened shortly after receiving doxycycline, raising concern for a drug reaction. Her condition progressed with significant ocular involvement requiring bilateral amniotic membrane grafting and subsequent immunosuppressive therapy. Investigations confirmed *Mycoplasma pneumoniae* infection, supporting a diagnosis most consistent with Mycoplasma-induced rash and mucositis (MIRM), although Stevens-Johnson syndrome (SJS) could not be fully excluded.

Following recovery, a structured allergy work-up was undertaken, including patch testing, intradermal testing and graded oral provocation in accordance with established guidance. All tests were negative, and the patient successfully tolerated amoxicillin, doxycycline and ibuprofen without adverse reactions.

This case demonstrates how a structured, multidisciplinary approach to drug allergy evaluation can facilitate de-labelling in patients with complex mucocutaneous presentations, restore access to first-line therapies and improve patient care.

## Introduction

A rash occurring after the introduction of a new medication is frequently attributed to drug allergy, and the diagnosis is rarely challenged through a structured clinical process. Consequently, patients accumulate multiple drug allergy labels over time, leading to significant restrictions in treatment options. This can be particularly detrimental when first-line therapies, such as antibiotics, are avoided, contributing to suboptimal treatment and antimicrobial resistance. Approximately one in 10 patients reports a penicillin allergy; however, up to 90% of these individuals can be safely de-labelled following formal assessment [1]. Inaccurate drug allergy labels not only pose a risk to patient safety but are also associated with prolonged hospital stays and increased healthcare costs [2].

Mycoplasma pneumoniae-induced rash and mucositis (MIRM) is an important differential diagnosis in patients presenting with severe mucocutaneous disease. MIRM is an immune-mediated syndrome associated with *Mycoplasma pneumoniae* infection, characterised by prominent multisite mucositis and limited cutaneous involvement [3]. In contrast, Stevens-Johnson syndrome (SJS) is typically a drug-induced, severe cutaneous adverse reaction characterised by widespread epidermal necrosis [4]. Given the overlap in clinical presentation, medications introduced during the prodromal illness may be incorrectly implicated, resulting in inappropriate long-term drug allergy labels [5].

We describe the case of a 23-year-old woman who presented with severe mucocutaneous disease initially suspected to be secondary to drug allergy. A structured diagnostic work-up enabled the de-labelling of multiple drug classes. The aim of this report is to demonstrate the value of a multidisciplinary approach to drug allergy evaluation following severe mucocutaneous disease with significant ocular involvement.

## Case presentation

A 23-year-old previously well female patient presented with severe bilateral conjunctivitis and painful oral ulceration, following a two-week prodrome of productive cough and coryza and a one-week history of fever. From day four of symptom onset, the patient was managing her cough and coryza with over-the-counter ibuprofen and paracetamol. On day 15 after symptom onset, she attended the local emergency department with fever, worsening productive cough, chest tightness, bilateral eye pain and mouth pain. A chest radiograph did not demonstrate convincing evidence of focal consolidation or collapse. However, the clinical history, examination findings and markedly elevated inflammatory markers were consistent with a possible respiratory tract infection. Oral doxycycline was prescribed for a suspected upper respiratory tract infection and topical chloramphenicol drops for bilateral conjunctivitis.

**Figure 1:**
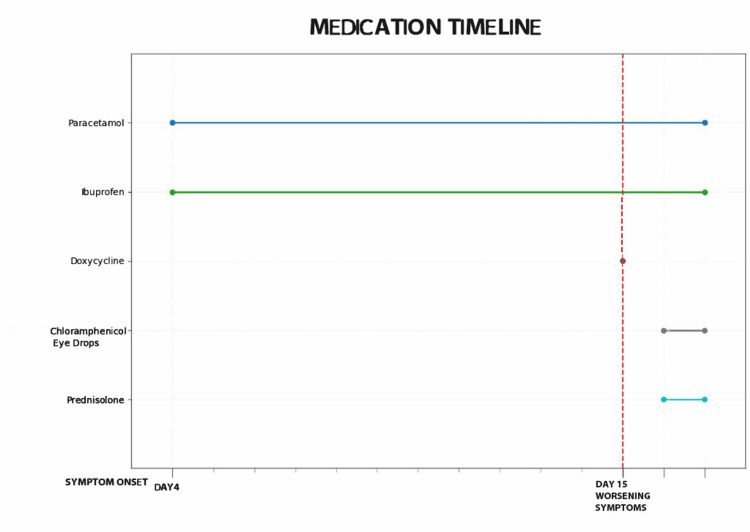
Timeline illustrating medication administration and clinical symptoms. Figure created using Med Timeline, available at: https://www.med-timeline.com. Data were manually entered by the author and arranged using the platform’s timeline generation function.

A few hours after a single dose of doxycycline, she developed a sensation of tongue and throat swelling, worsening oral mucosal pain, as well as lip and face swelling, prompting admission to her local hospital (Figure 1). Examination revealed conjunctival redness and marked oral mucositis. No other areas of skin were affected. Thirty milligrams of oral prednisolone daily was commenced, in addition to the continuation of chloramphenicol eye drops. The day after admission, she experienced blurring of her vision. Due to severe ocular deterioration, she was transferred urgently to a tertiary hospital and had bilateral amniotic membrane grafts. Topical ocular treatment included hourly dexamethasone 0.1% drops and sodium hyaluronate with trehalose drops, as well as levofloxacin 0.5% eye drops four times daily. Despite initial intervention, she developed cicatrising conjunctivitis and symblepharon formation, requiring repeat bilateral amniotic grafts, eight days post initial procedure. On day 11 post bilateral amniotic membrane grafts, she was prescribed intravenous immunoglobulins (1 g/kg for two days) to manage the inflammation. 

Extensive infective screen tests were performed, which detected the presence of *Mycoplasma pneumoniae* IgM and IgG (Table 1). The clinical features and chronology were felt to favour MIRM. However, given the exposure to multiple medications during the acute illness, SJS could not be completely excluded. Oral and ocular mucosal symptoms were already established prior to doxycycline administration, and the absence of widespread cutaneous involvement further supported MIRM as the most likely diagnosis.

**Table 1:** Summary of infective screen and serological findings

Test	Result
Mycoplasma IgG Antibody	Detected
*Mycoplasmoides pneumoniae* IgM	Detected
Epstein-Barr Virus capsid IgG	Detected at low levels
Epstein-Barr Virus capsid IgM	Equivocal
Hepatitis B screen	Negative
Hepatitis C screen	Negative
Hepatitis E screen	Negative
Human Immunodeficiency Virus 1+2 total antibodies	Negative
*Neisseria gonorrhoea* PCR	Negative
Herpes Simplex Virus 1+2 DNA	Negative
Varicella Zoster Virus DNA and IgG	Negative
Cytomegalovirus IgG and IgM antibodies	Negative
Quantiferon Tuberculosis	Negative

**Figure 2:**
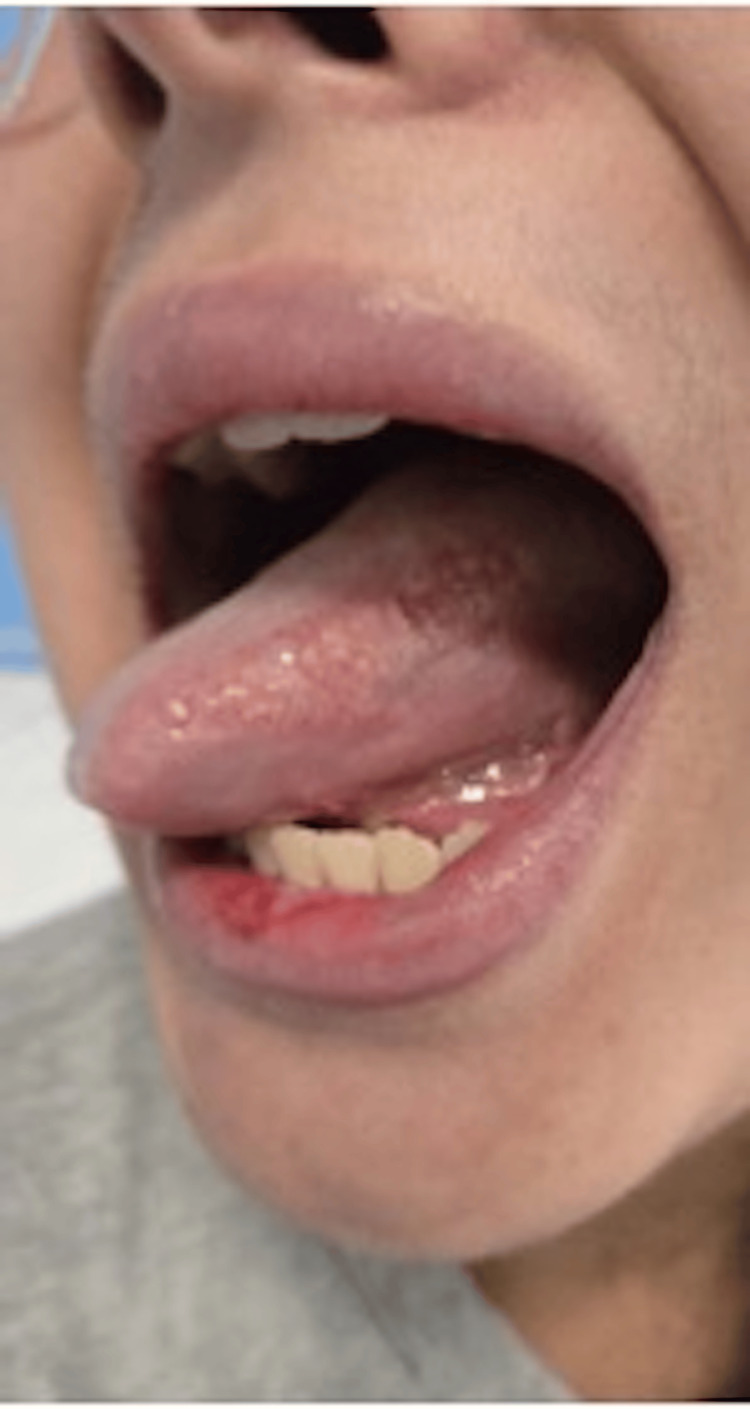
Clinical image demonstrating oral mucosal erosions and haemorrhagic crusting consistent with mucosal involvement in Mycoplasma-induced rash and mucositis (day 5 of treatment)

Her mucosal symptoms gradually improved with oromucosal erosions and ulcers beginning to heal, whilst hemorrhagic crusting began to resolve (Figure 2). She was discharged on day 16 after hospitalisation to complete a tapering regimen of prednisolone 30 mg (reducing by 10 mg every seven days) alongside the eye drops. Six days after discharge, she was reviewed in ophthalmology outpatient, with improving ocular surface inflammation. 

The patient had been labelled as allergic to amoxicillin since childhood and was additionally advised to avoid tetracyclines and non-steroidal anti-inflammatory drugs (NSAIDs) following discharge, resulting in significant restriction of future therapeutic options. She was therefore referred to the regional drug allergy service for formal evaluation. Allergy testing commenced four months after administration of intravenous immunoglobulin and three months following completion of oral prednisolone, thereby minimising the potential impact of immunomodulatory therapy on test performance.

Prior to drug testing, the patient underwent detailed counselling regarding the risks and limitations of patch testing, intradermal testing and drug provocation testing, including the possibility of false-positive, false-negative and sensitisation reactions. 

Testing was commenced at low dose with an initial drug patch test at 1% active concentration on the upper arm, to enable patient monitoring for reactions (Table 2). Reviews were performed at day 2 and then delayed reading on day 7, which were negative. Therefore, further drug patch tests at 10% active concentration were performed on the contralateral arm with a similar review schedule (Table 2). Follow-up was conducted virtually via telephone consultation, with confirmation using patient-submitted photographs.

**Table 2:** Epicutaneous drug patch tests performed. Patch test with allergens from Chemotechnique (Chemotechnique Diagnostics AB, Vellinge, Sweden) on Scanpor® medical adhesive tape (Norgesplaster AS, Vennesla, Norway) unless specified; * = compounded from oral tablets. pet: petrolatum

Drug patch test		
Week 1	Day 2	Day 7
Doxycycline 1% pet*	-	-
Ibuprofen 1% pet*	-	-
Diclofenac 1% pet*	-	-
			Week 2		
Doxcycyline 10% pet	-	-
Ibuprofen 10% pet	-	-
Diclofenac 5% pet	-	-
Week 3		
Amoxicillin 10% pet	-	-
Flucloxacillin 10% pet	-	-
Doxycycline 30% pet*	-	-

**Figure 3:**
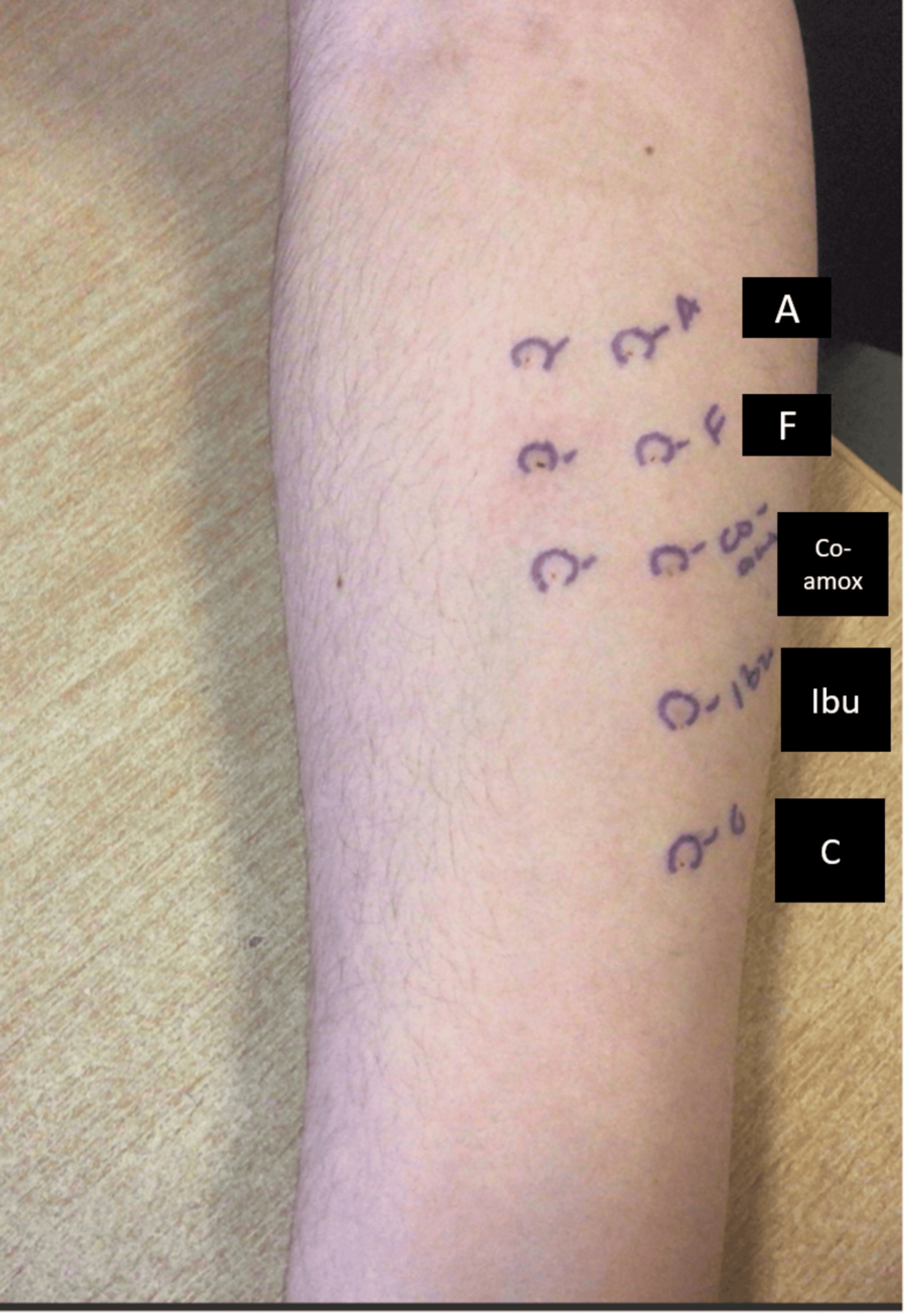
Intradermal drug test Intradermal drug test results for amoxicillin (A, 2 mg/mL), flucloxacillin (F, 2 mg/mL), co-amoxiclav (Co-amox, 2 mg/mL), and ibuprofen (Ibu, 0.1 mg/mL). Saline (C) served as the negative control.

Following negative drug patch tests, intradermal testing was performed using standard concentrations, with delayed readings taken at day 7 (Table 3, Figure 3).

**Table 3:** Intradermal drug tests performed Intradermal test from sterile intravenous formulations.

Intradermal test		
Week 3	Day 2	Day 7
Amoxicillin 2 mg/ml	-	-
Flucloxacillin 2 mg/ml	-	-
Co-amoxiclav 2 mg/ml	-	-
Ibuprofen 0.1 mg/ml	-	-
Week 4		
Amoxicillin 20 mg/ml	-	-
Flucloxacillin 20 mg/ml	-	-
Co-amoxiclav 20 mg/ml	-	-

Following negative drug patch tests, intradermal testing was performed using standard concentrations, with delayed readings taken at day 7 (Table 3). Concentrations were as per the European Network for Drug Allergy and the European Academy of Allergy and Clinical Immunology (EDNA/EAACI) [6]. 

The decision to proceed with drug provocation testing was made following a multidisciplinary review. Despite the severity of the initial presentation and its associated ocular complications, the chronology of events was considered inconsistent with SJS. Oral and ocular mucosal symptoms pre-dated doxycycline exposure, and the overall clinical picture was felt to be more consistent with the progression of MIRM. Following careful consideration of the potential risks and benefits, and after providing informed consent, the patient proceeded with oral drug provocation testing. The oral drug provocation test was initiated at one-tenth of the usual therapeutic dose (Table 4) with escalation to the full dose on day 2 and continued for a total of three days. The patient's PEN-FAST score (Penicillin allergy, Five years or less since the reaction, Anaphylaxis/angioedema, Severe reaction, and Treatment required) was low (<3), corresponding to an estimated 5% or lower risk of true penicillin allergy, supporting further allergy assessment [7]. Doxycycline and ibuprofen were subsequently subjected to oral drug provocation testing, which was completed without adverse effects. 

**Table 4:** Oral provocation drug tests performed BD: twice daily; TDS: three times daily

Provocation test			
Week 5	Day 0	Day 2	Days 4 to 7
Amoxicillin	250 mg single dose	250 mg TDS	500 mg TDS
Week 6			
Doxcycline	20 mg single dose	20 mg BD	100 mg BD
Week 7			
Ibuprofen	20 mg single dose	200 mg single dose	200 mg TDS

The patient remained under regular ophthalmology surveillance throughout the de-labelling process, with no evidence of recurrent ocular inflammation, deterioration in visual status, or recurrence of mucosal symptoms. The patient was therefore confirmed to be tolerant of all three medication classes, and her medical records were updated accordingly.

## Discussion

MIRM and SJS are now recognised as distinct clinical entities; however, differentiating between them remains challenging in clinical practice, particularly when medications are introduced during the prodromal phase of a respiratory illness [5]. Severe mucosal and ocular involvement may raise concern for a drug-induced severe cutaneous adverse reaction, even in the absence of significant cutaneous findings, potentially leading to inappropriate attribution of symptoms to recently prescribed medications. Consequently, patients may acquire inaccurate drug allergy labels that persist long after the acute illness has resolved, despite a lack of evidence for true drug hypersensitivity.

Drug allergy labels, especially those related to antibiotics, frequently persist into adulthood based on unclear histories of previous reactions. As a result, therapeutic options are unnecessarily restricted in many individuals. Studies have shown that between 8% and 25% of patients report a penicillin allergy; however, approximately 90% of these individuals are not truly allergic when formally assessed through structured de-labelling protocols [8,9].

Given the growing concern over multi-resistant organisms, antibiotic allergy de-labelling has become a critical aspect of patient management [10,11]. The British Society for Allergy and Clinical Immunology (BSACI) has developed guidelines aimed at standardising the de-labelling process, with a particular focus on penicillin allergy, enabling non-specialists to implement this approach effectively [12]. These guidelines emphasise the identification and classification of patients into different risk groups, as well as the use of drug provocation testing [12, 6]. BSACI focuses on identifying patients with non-severe reaction histories, who are then eligible for direct oral amoxicillin challenges in a monitored setting [12]. Patients with complex histories or suspected severe delayed reactions, such as the patient described in this report, are referred to allergy specialists for skin prick testing, intradermal testing and, finally, oral provocation [12].

Not only does allergy de-labelling increase treatment options, but it also has economic benefits for the healthcare system. Patients with penicillin allergy labels are more likely to receive alternative, often broader-spectrum antibiotics, which are typically more expensive and associated with longer hospital stays [2]. This contributes to increased overall healthcare costs, driven by both drug expenditure and resource utilisation [2]. In contrast, structured de-labelling pathways enable the use of first-line, cost-effective therapies and have been shown to be safe and scalable in clinical practice, supporting both financial sustainability and antimicrobial stewardship [1].

Investigations in this patient enabled access to three widely used classes of drugs, which would otherwise have resulted in significant limitation of antimicrobial and analgesic options. Although the benefits of drug allergy de-labelling are well established, several challenges remain. Given the large number of individuals carrying allergy labels, systematically identifying appropriate candidates for de-labelling represents a considerable logistical and clinical undertaking. Additionally, the de-labelling process can be time-consuming and resource-intensive for both clinicians and patients, posing barriers to implementation in routine practice [13]. As with most diagnostic tests, a negative skin test does not rule out the possibility of a false-negative outcome. It is therefore important to risk-stratify patients prior to performing the drug provocation test [14]. From the patient's perspective, the process may also be a source of anxiety. A study explored the experiences of families whose children underwent graded oral drug challenges, highlighting that many allergy labels had been applied following the appearance of a rash, often without adequate investigation to confirm a true allergy [15]. These findings emphasise the importance of careful assessment prior to labelling, recognising that while drug reactions and allergies are important differentials, premature labelling without appropriate evaluation may lead to long-term consequences for patient care.

In this case, the patient underwent a structured drug de-labelling process, beginning with drug patch testing at low concentration, followed by intradermal testing, and ultimately oral provocation. One could argue that, given the low likelihood of drug hypersensitivity, supported by symptom onset preceding drug initiation and clinical features consistent with MIRM, an oral challenge could have been performed without the need for epicutaneous testing. However, patients can develop sensitisation to drugs administered during the illness, and performing sequential skin tests in the absence of a visible rash can help build patient confidence before proceeding to an oral challenge [16]. Patients recovering from severe illness may be understandably hesitant to reintroduce drugs previously suspected as triggers. While cautious, initial low-concentration skin testing can help build trust and minimise risk before moving to a provocation test.

This case highlights the importance of a structured approach to drug allergy evaluation in patients presenting with severe mucocutaneous disease. While the clinical features and chronology were considered more consistent with MIRM than SJS, the overlap between these conditions can make the distinction challenging. 

A distinguishing feature of this case was the successful implementation of a structured multidisciplinary de-labelling pathway following severe mucocutaneous disease with significant ocular involvement requiring bilateral amniotic membrane grafting. Close collaboration between dermatology, ophthalmology and allergy specialists supported a diagnosis most consistent with MIRM, despite the complexity and severity of the presentation. Formal allergy assessment subsequently enabled the safe removal of multiple drug allergy labels, restoring access to first-line therapies and avoiding unnecessary restrictions on future treatment options. In addition to restoring access to first-line therapies, accurate de-labelling supports antimicrobial stewardship by reducing unnecessary use of broader-spectrum alternative antibiotics, which have been associated with antimicrobial resistance and an increased risk of infection with drug-resistant organisms [17]. This case highlights the value of multidisciplinary management in preventing unnecessary long-term allergy labelling and its downstream consequences.

## Conclusions

This case highlights the importance of a structured approach to drug allergy evaluation following severe mucocutaneous reactions. Although the clinical presentation was most consistent with MIRM, overlap with drug-induced SJS remains a recognised diagnostic challenge. Through multidisciplinary assessment and formal allergy testing, multiple drug allergy labels were successfully removed, restoring access to antibiotics and other first-line therapies. Furthermore, this case demonstrates that even in the context of severe ocular involvement requiring bilateral amniotic membrane grafting, careful clinical reassessment and specialist allergy evaluation can facilitate appropriate de-labelling, preserve future treatment options and support antimicrobial stewardship.
